# Caregiver burden in Bardet-Biedl syndrome: findings from the CARE-BBS study

**DOI:** 10.1186/s13023-023-02692-8

**Published:** 2023-07-07

**Authors:** Elizabeth Forsythe, Usha G. Mallya, Min Yang, Caroline Huber, Mary Lynn Cala, Alexandra Greatsinger, Ella Hagopian, Jeremy Pomeroy, Andrea M. Haqq

**Affiliations:** 1 University College London Great Ormond Street Institute of Child Health, London, WC1N 1EH UK; 2 Rhythm Pharmaceuticals, Inc., Boston, MA USA; 3 Analysis Group, Inc., Boston, MA USA; 4 Marshfield Clinic Research Institute, Marshfield, WI USA; 5 Division of Pediatric Endocrinology, University of Alberta, Edmonton, AB Canada

**Keywords:** Bardet-Biedl syndrome, Caregiver burden, Hyperphagia, Obesity, Personal strain, Work productivity

## Abstract

**Background:**

Bardet-Biedl syndrome (BBS) is a rare, genetically heterogeneous obesity syndrome associated with hyperphagia. Given the early onset of BBS symptoms in childhood and multifaceted complications, this study aimed to quantify the caregiver burden associated with BBS.

**Methods:**

A cross-sectional, multi-country survey of caregivers from the United States (US), United Kingdom (UK), Canada, and Germany was designed to quantify the extent of caregiver burden associated with obesity and hyperphagia symptoms (i.e., uncontrollable hunger) among patients with BBS.

**Results:**

A total of 242 caregivers across the four countries met the inclusion criteria and completed the survey. The mean (standard deviation [SD]) age of the caregivers was 41.9 (6.7) years, and the mean (SD) age of individuals with BBS in their care was 12.0 (3.7) years. Hyperphagia contributed to a BBS diagnosis in 230 of 242 individuals (95.0%). On average, caregivers used eight different weight management approaches for those in their care and expressed a strong desire for more effective weight management methods. Based on the *Impacts of Hyperphagia: Caregiver version*, patients’ hyperphagia had a moderate-to-severe impact on caregiver mood (56.6%), sleep (46.6%), and relationships (48.0%). Caregivers reported experiencing a high level of personal strain (mean [SD], 17.1 [2.9]) and family impact (mean [SD] score, 26.0 [3.8]) due to BBS, as measured by the *Revised Impact on Family Scale*. Among caregivers in the workforce, there also was high impairment in total work productivity (mean [SD], 60.9% [21.4%]) due to caring for patients with BBS according to the *Work Productivity and Activity Impairment*. More than half (53%) of the caregivers reported spending over 5,000 out-of-pocket in local currency for medical expenses for the patient with BBS in their care.

**Conclusions:**

Obesity and hyperphagia have negative impacts on the lives of caregivers of patients with BBS. The burden is demonstrated to be multifaceted, with various components that may interact with and confound each other, including intensive weight management efforts, productivity loses, impaired family dynamics and out-of-pocket medical expenses.

**Supplementary Information:**

The online version contains supplementary material available at 10.1186/s13023-023-02692-8.

## Introduction

Bardet-Biedl syndrome (BBS) is a rare, genetically heterogeneous syndrome that affects approximately 4000–5000 patients in the United States (US), with an incidence rate of 1:100,000-1:140,000 in North America and 1:125,000-1:160,000 in Europe [1–3]. BBS is a ciliopathy, a class of disorders associated with genetic mutations that result in abnormal formation or function of cilia [4]. As a result, it is characterized by multi-systemic clinical features and complications that often begin to appear during childhood, including retinal dystrophy, postaxial polydactyly, obesity, genital anomalies, renal anomalies, and learning disabilities [3, 5]. Severe early-onset obesity and pathological insatiable hunger (hyperphagia) are two key characteristic manifestations of this rare genetic disorder. Based on data from the Clinical Registry Investigating BBS (CRIBBS), 70% of patients with BBS have obesity relative to only 20% of the general population [6, 7].

Historically, obesity associated with BBS has been treated symptomatically with a focus on the management of diabetes, hypertension, and metabolic syndrome to delay the onset of secondary complications among these patients [5, 8]. Given the negative effects of obesity and hyperphagia on health and quality of life, weight management and control of hyperphagia are two of the key goals for patients with BBS. However, this is challenging because caregivers must implement strict environmental controls, such as supervising children around food, securing food sources, reducing energy intake, and adhering to meal schedules [6]. While controlling caloric intake, these strategies often fail to address the persistent underlying hyperphagia [6].

The hyperphagia attributes associated with BBS, particularly food-seeking behavior, are often characterized as relentless and overwhelming and can result in a substantial burden on families and caregivers that negatively affects their well-being [9]. Caregivers may also be at risk of potentially increased isolation from their communities and/or feelings of loneliness due to the all-consuming need to care for the patient, and the social stigmatization of having a child dealing with obesity and abnormal food seeking behaviors. The net impact of this burden translates to impaired work productivity, and an increased financial burden [10, 11]. However, the challenges facing caregivers and families of patients with BBS in everyday life are not well-quantified, which impairs the development of optimal strategies to sustain caregivers in their vital roles of supporting patients with BBS. To that end, this survey study, CARE-BBS (CAREgiver Burden in Bardet-Biedl Syndrome), aimed to quantify the extent of the physical, emotional, and financial toll on caregivers providing care for patients with BBS.

## Methods

### Study population

A cross-sectional, multi-country survey was designed to collect data from adult caregivers of patients with BBS who live with obesity and hyperphagia. Caregivers were recruited from the US, United Kingdom (UK), Canada, and Germany through a market research panel if they cared for a patient with BBS for ≥ 6 months and were able to read and understand the local language of their country. Participating caregivers were required to complete a short screener to confirm they met the study inclusion and exclusion criteria. Inclusion criteria were all caregiver-reported and included the patient having BBS and obesity. The obesity criteria could be met via caregiver-reported the patient currently having obesity or ever having a weight in the ≥ 95th percentile for the patient’s age and sex. Professional caregivers (i.e., those paid for their time to care for the patient with BBS) and caregivers of patients who were enrolled in a clinical trial at the time of the survey or during the preceding 6 months were excluded from the study. Caregivers who provided informed consent and met all inclusion criteria were invited to complete the full survey and received an honorarium for their time to complete the survey. This study was conducted in accordance with the Helsinki Declaration of 1964 and its later amendments and was granted an exemption from a full review by the US Pearl Independent Review Board.

### Survey components

The survey was designed to collect information on patient characteristics as well as caregivers’ socio-demographics, medical history, and burden of caregiving. Specifically, caregiver burden was characterized across several domains, including the impact of caregiving on the caregiver’s professional work and productivity, activities of daily life, physical and mental health, and financial stress, specifically with regards to financial costs associated with patients’ medical care, weight management, and caregiver-reported expenses related to patient care. The specific measures are as follows:

#### Impacts of hyperphagia© (IoH): caregiver version

The newly developed *Impacts of Hyperphagia: Caregiver version* contains 5 items measuring the impact of hyperphagia on the daily life of the caregiver regarding sleep, mood/emotions, work, leisure/recreational activities and relationships using a 4-point agreement scale (“Not at all”, “A little”, “Moderately”, “A great deal”). An overall score was generated by summing the scores across all items with a score range from 0 to 15, where higher scores indicate greater impacts of hyperphagia.

#### Work productivity and activity impairment (WPAI)–obesity associated with BBS: caregiver

The WPAI questionnaire was adapted to measure the impact of caregiving on productivity (e.g., hours missed from work and actual hours worked) and impairment on work and regular activities due to caregiving for someone with BBS. The WPAI has 6 items and a recall period of “the past 7 days.” Items 5 and 6 that measure impacts on work and impairment of activities utilize an 11-point numeric rating scale (NRS) (0 “Health problem has no effect on my work/daily activities” to 10 “Health problem completely prevented me from working/doing my daily activities”). The WPAI produces 4 scores based on the following outcomes: absenteeism (percentage of work time missed), presenteeism (percentage of impairment while working, total productivity impairment (percentage of overall work impairment), and total activity impairment (percentage of activity impairment). Scores range from 0–100% whereby a higher percentage indicates greater work or activity impairment [12].

#### Patient-reported outcomes measurement information system® (PROMIS) scale v1.2–global health: adult

The PROMIS Scale v1.2–Global Health has 10 items assessing an adult’s overall health. This instrument generates a Global Mental Health score and a Global Physical Health score. All items except one use 5-point Likert scales (5 “Excellent” to 1 “Poor”; 5 “Completely” to 1 “Not at all”; 5 “Never” to 1 “Always”, and 5 “None” to 1 “Very severe”). One item that measures pain is on an 11-point NRS whereby 0 represents “No pain” and 10 represents “Worst pain imaginable”. A T-score was calculated using response pattern scoring; a higher T-score represents better overall health. T-scores can range between 16.2 and 67.7 for physical health and 21.2–67.6 for mental health [13].

#### Revised impact on family scale© (RIOFS)

The RIOFS has 15 items assessing a family member’s perception of the effect of a patient’s chronic condition on family life. The instrument has been shown to have strong face validity and favorable psychometric evaluations, including construct validity [14]. The RIOFS generates a total score using a 4-point Likert scale (“Strongly Agree”, “Agree”, “Disagree”, “Strongly Disagree”) whereby higher scores indicate that the patient’s chronic condition has a greater impact on family life.

Other measures assessed in the CARE-BBS study included the Symptoms of Hyperphagia: Caregiver Version, the PROMIS Parent Proxy Scale v1.0—Global Health, and the IWQOL-Kids: Parent-Proxy, which are reported elsewhere as they are beyond the scope of the current analysis.

### Statistical analyses

Data were pooled across the four countries, and the demographic and medical history of caregivers were described overall and by country. Responses underwent a quality check to assess internal logic and quality of responses, and responses that failed to meet quality checks were excluded from analyses. Instruments were scored, including the total score and domain scores, where applicable, and descriptively summarized overall and by country. Given the early onset of BBS symptoms in many patients, the WPAI questionnaire and PROMIS Scale outcomes were stratified by the age of the patient with BBS (age groups: < 6, 6–11, 12–17, 18 +) to understand if the age of the patient with BBS had a differential impact on caregivers’ ability to work and their general health. Means and standard deviations (SDs) were reported for continuous variables, while counts and percentages were reported for categorical variables. R 3.6.3 (R Core Team, 2020) was used for all data analyses.

## Results

### Study population characteristics

The final study sample included a total of 242 caregivers who met the eligibility criteria, completed the survey, and passed quality checks, among whom 60 were from the US, 59 were from the UK, 62 were from Canada, and 61 were from Germany. Two caregivers who met the inclusion criteria but failed the quality check due to logically inconsistent responses were excluded from the analysis. The median (SD) age of caregivers was 41.9 (6.7) years with 54% male, and 93% being a parent of the patient (52% being the father and 42% being the mother). The majority of caregivers were married or in a domestic partnership (86%). Nearly all caregivers (98%) reported that there was another person assisting with caregiving responsibilities, most often another parent (73%). Caregivers generally reported being in good health, with a small proportion having an eating disorder, anxiety, high blood pressure, high cholesterol, or a sleep disorder (all < 10%). More than 80% reported having household income ≥ 75,000 in local currency. Caregiver characteristics were generally similar across country of residence, though Germany had a notably higher proportion of male participants (62.3%) and a slightly lower proportion of participants who reported being married or in a domestic partnership (78.7%) than other countries (Table 1).

**Table 1:** Demographics and Medical History of Caregivers by Country

	Overall N = 242	Canada N = 62	Germany N = 61	UK N = 59	US N = 60
Demographics					
Age (years), mean ± SD (median)	41.9 ± 6.7 (42)	42.9 ± 6.7 (42)	42.1 ± 7.7 (43)	40.1 ± 7.9 (41)	42.4 ± 3.6 (43)
Sex, n (%)					
Male	131 (54.1)	29 (46.8)	38 (62.3)	34 (57.6)	30 (50.0)
Female	111 (45.9)	33 (53.2)	23 (37.7)	25 (42.4)	30 (50.0)
Married or in a domestic partnership, n (%)	209 (86.4)	52 (83.9)	48 (78.7)	52 (88.1)	57 (95.0)
Highest education attainment (top 4), n (%)					
High school diploma/equivalent or lower	16 (6.6)	1 (1.6)	12 (19.7)	3 (5.1)	-
Some college/university or Associate’s degree	56 (23.1)	20 (32.3)	12 (19.7)	17 (28.8)	7 (11.7)
College or university graduate/bachelor’s degree	107 (44.2)	27 (43.5)	25 (41.0)	25 (42.4)	30 (50.0)
Advanced degree	63 (26.0)	14 (22.6)	12 (19.7)	14 (23.7)	23 (38.3)
Household income (in local currency), n (%)					
< 75,000	33 (13.6)	5 (8.1)	17 (27.9)	10 (16.9)	1 (1.7)
≥ 75,000	208 (86.0)	57 (91.9)	43 (70.5)	49 (83.0)	59 (98.3)
Prefer not to say	1 (0.4)	-	1 (1.6)	-	-
Relationship to person with BBS, n (%)					
Mother	101 (41.7)	30 (48.4)	21 (34.4)	20 (33.9)	30 (50.0)
Father	125 (51.7)	28 (45.2)	34 (55.7)	33 (55.9)	30 (50.0)
Other	16 (6.6)	4 (6.5)	6 (9.8)	6 (10.2)	-
Others responsible for care of person with BBS, n (%)					
Parent	176 (72.7)	43 (69.4)	46 (75.4)	46 (78.0)	41 (68.3)
Grandparent	29 (12.0)	7 (11.3)	10 (16.4)	7 (11.9)	5 (8.3)
Other	32 (13.2)	8 (12.9)	9 (14.8)	7 (11.9)	8 (13.3)
No others are responsible	43 (17.8)	10 (16.1)	11 (18.0)	8 (13.6)	14 (23.3)
Medical History					
Currently receiving treatment for condition (top 5), n (%)					
Eating disorders	19 (7.9)	1 (1.6)	4 (6.6)	11 (18.6)	3 (5.0)
Anxiety disorders	13 (5.4)	1 (1.6)	4 (6.6)	3 (5.1)	5 (8.3)
High blood pressure	12 (5.0)	1 (1.6)	5 (8.2)	3 (5.1)	3 (5.0)
High cholesterol	12 (5.0)	2 (3.2)	2 (3.3)	3 (5.1)	5 (8.3)
Sleep disorders	10 (4.1)	4 (6.5)	2 (3.3)	2 (3.4)	2 (3.3)

BBS: Bardet-Biedl Syndrome; SD: standard deviation

The mean (SD) age of patients with BBS was 12.0 (3.7) years, with 63% between ages 12 and17 and 64% males. The mean (SD) time since BBS diagnosis was 4.2 (2.8) years, and obesity and hyperphagia contributed to a BBS diagnosis in the majority of the patients (95%). The majority (85%) of patients in the sample were considered to have obesity at the time of the survey (95th percentile or above for pediatric patients, and BMI of 30 or higher for adult patients), followed by being overweight (11%) (pediatric patients in the 85th to < 95th percentile, or BMI of 25 to < 30 for adult patients); 10 patients (4%) were considered to have normal weight. About a third (38%) of caregivers reported that a genetic test related to BBS was performed on the patient with BBS, and almost two-thirds (63%) of these caregivers reported that a mutation was found in the test results. Patient characteristics were similar across country of residence.

### Direct impacts due to patients’ hyperphagia

On average, caregivers reported employing eight strategies at the time of the survey to manage the weight of the patient with BBS (Additional file 1). The average number of strategies reported was similar across country of residence, with a maximum average number of strategies of 8.8 in the US and a minimum of 7.4 average strategies in the UK. While caregivers reported satisfaction with existing weight management approaches (mean (SD) score of 7.4 (1.6) on a scale of 0 “lowest satisfaction” to 10 “highest satisfaction”), they felt that having new effective approaches to manage weight was highly important (mean (SD) score of 7.8 (1.4) on a scale of 0 “lowest importance” to 10 “highest importance”).

The majority of caregivers (~ 90%) reported that the patient’s hyperphagia had at least some negative impact on the caregiver’s sleep, mood/emotions, work, leisure activities and/or relationships with family or friends. Around half of caregivers reported the impact was “moderate” or “a great deal” in the following domains: sleep over the past 7 days (57%), mood or emotions (57%), work (53%), leisure or recreational activities (53%), and relationships with family or friends (48%). More caregivers in the UK reported an impact on sleep over the past 7 days (71%), and fewer caregivers in Canada reported an impact on work (34%) and on relationships with friends and family (29%) versus the overall caregiver sample (Table 2).

**Table 2:** IoH© – Caregivers by Country

	Overall N = 242	Canada N = 62	Germany N = 61	UK N = 59	US N = 60
Average summary scale scores (Range: 0–15) mean ± SD	7.8 ± 3.2	6.5 ± 2.5	7.8 ± 3.4	8.7 ± 3.5	8.4 ± 2.7
During the past 7 days to what extent did the person in your care’s hunger negatively affect your…					
Sleep? n (%)					
Not at all	23 (9.5)	9 (14.5)	5 (8.2)	2 (3.4)	7 (11.7)
A little	82 (33.9)	22 (35.5)	25 (41.0)	15 (25.4)	20 (33.3)
Moderately	94 (38.8)	26 (41.9)	21 (34.4)	26 (44.1)	21 (35.0)
A great deal	43 (17.8)	5 (8.1)	10 (16.4)	16 (27.1)	12 (20.0)
Mood or emotions? n (%)					
Not at all	28 (11.6)	9 (14.5)	9 (14.8)	6 (10.2)	4 (6.7)
A little	77 (31.8)	25 (40.3)	15 (24.6)	17 (28.8)	20 (33.3)
Moderately	97 (40.1)	23 (37.1)	24 (39.3)	26 (44.1)	24 (40.0)
A great deal	40 (16.5)	5 (8.1)	13 (21.3)	10 (16.9)	12 (20.0)
Work? n (%)					
Not at all	33 (13.6)	10 (16.1)	9 (14.8)	7 (11.9)	7 (11.7)
A little	80 (33.1)	31 (50.0)	19 (31.1)	14 (23.7)	16 (26.7)
Moderately	92 (38.0)	20 (32.3)	23 (37.7)	27 (45.8)	22 (36.7)
A great deal	37 (15.3)	1 (1.6)	10 (16.4)	11 (18.6)	15 (25.0)
Leisure or recreational activities? n (%)					
Not at all	34 (14.0)	11 (17.7)	11 (18.0)	8 (13.6)	4 (6.7)
A little	80 (33.1)	22 (35.5)	19 (31.1)	17 (28.8)	22 (36.7)
Moderately	81 (33.5)	22 (35.5)	23 (37.7)	15 (25.4)	21 (35.0)
A great deal	47 (19.4)	7 (11.3)	8 (13.1)	19 (32.2)	13 (21.7)
Relationships with family or friends? n (%)					
Not at all	40 (16.5)	15 (24.2)	7 (11.5)	10 (16.9)	8 (13.3)
A little	86 (35.5)	29 (46.8)	23 (37.7)	17 (28.8)	17 (28.3)
Moderately	83 (34.3)	18 (29.0)	21 (34.4)	17 (28.8)	27 (45.0)
A great deal	33 (13.6)	-	10 (16.4)	15 (25.4)	8 (13.3)

IoH: Impacts of Hyperphagia; SD: standard deviation

### Impacts on ability to work and general health

**Fig. 1:**
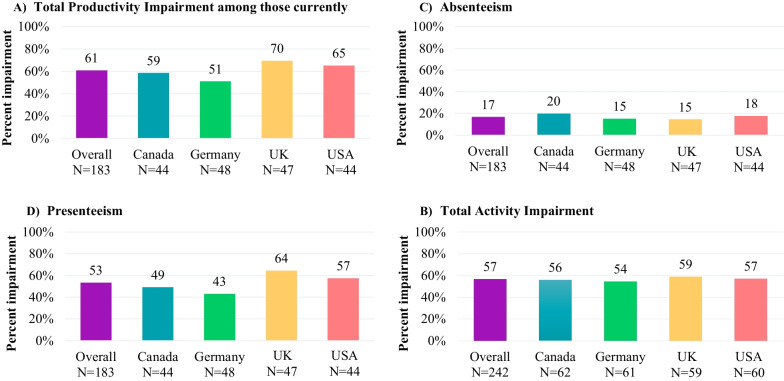
WPAI—BBS Caregiver by Country (Range: 0–100%). BBS: Bardet-Biedl Syndrome; WPAI: Work Productivity and Activity Impairment

Since they started caring for a patient with BBS, more than half the caregivers reported that it affected their ability to work, including 20% who reduced their work hours, 19% who temporarily stopped working or went on leave, and 15% who permanently stopped working or retired early. Results were heterogenous across countries. Compared to the overall sample, a higher proportion of UK caregivers reported having to switch jobs (9% vs. 18%), temporarily stop working (19% vs. 29%) and reduce work hours (20% vs. 29%), while a higher proportion of German caregivers reported early retirement (20%; Additional file 2). Three-quarters (76%) of caregivers reported working at the time of the survey: 59% were full-time employees, followed by 14% who were part-time employees and 5% who were self-employed (Additional file 3). Caregivers who were working at the time of the survey reported substantial impairment in productivity on the WPAI questionnaire, with an average total productivity impairment of 61%, absenteeism of 17%, and presenteeism of 53%; the total activity impairment was also substantial at 57%. The extent of total productivity impairment and presenteeism were highest among caregivers in the UK (70% and 64%) and lowest among caregivers in Germany (51% and 43%, respectively; Fig. 1**)**. When stratified by patient age, caregivers of patients in the < 6 and 6–11 age groups reported being most impacted as it related to their ability to work and perform regular daily activities.

**Fig. 2:**
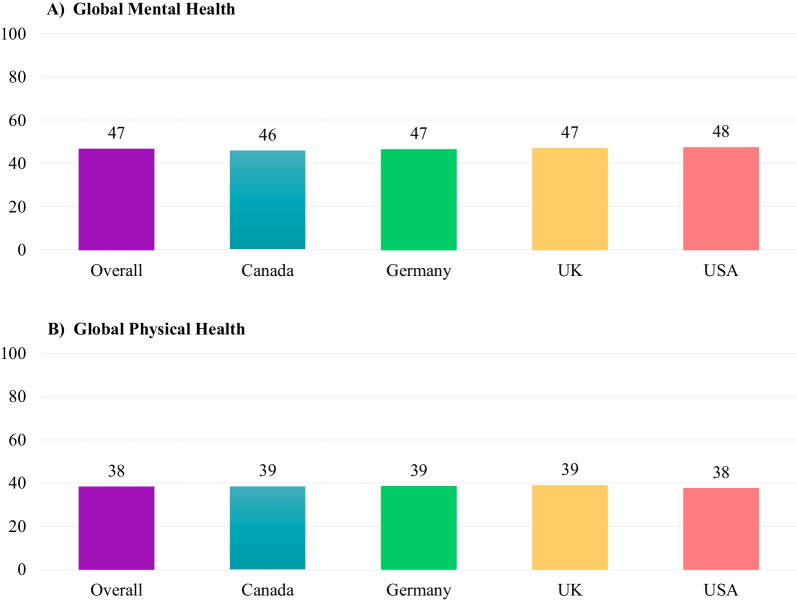
PROMIS® Scale—BBS Caregiver by Country. BBS: Bardet-Biedl Syndrome; PROMIS: Patient-Reported Outcomes Measurement Information System

Though caregivers reported a mean (SD) score of 46.8 (6.5) regarding their own mental health on the PROMIS Scale, which is comparable to the general population, they reported poorer physical health with a mean (SD) score of 38.4 (3.5; Fig. 2). Nearly half (49%) of the caregivers considered their overall health to be “very good” or “excellent”, and a similar proportion (48%) considered they carried out their usual social activities and roles “very good” or “excellent.” Self-rated overall and mental health PROMIS scores were similar across countries.

When stratified by patient age, the PROMIS Global Mental Health score for caregivers was lowest for those who cared for patients in the < 6 and 18 + age groups, with mean (SD) ratings of 42.6 (6.8) and 44.0 (7.0), respectively. Mental health ratings of caregivers were slightly better in the 6–11 and 12–17 age groups, with mean (SD) scores of 48.3 (6.4) and 46.5 (6.4), respectively. The PROMIS Global Physical Health score stratified by patient age was consistently relatively low in the 6–11, 12–17, and 18 + age groups, with average ratings of 38–39.

### Impacts on family

**Fig. 3:**
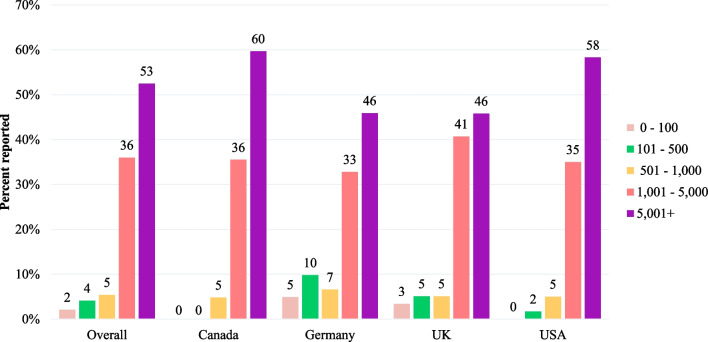
Out-of-Pocket Medical Expenses for Patients with BBS over the Past 12 Months by Country in Local Currency. BBS: Bardet-Biedl Syndrome

**Fig. 4:**
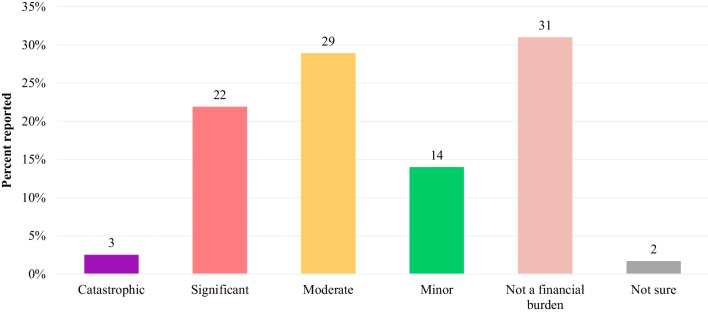
Financial Burden of Caring for Patients with BBS. BBS: Bardet-Biedl Syndrome

**Fig. 5:**
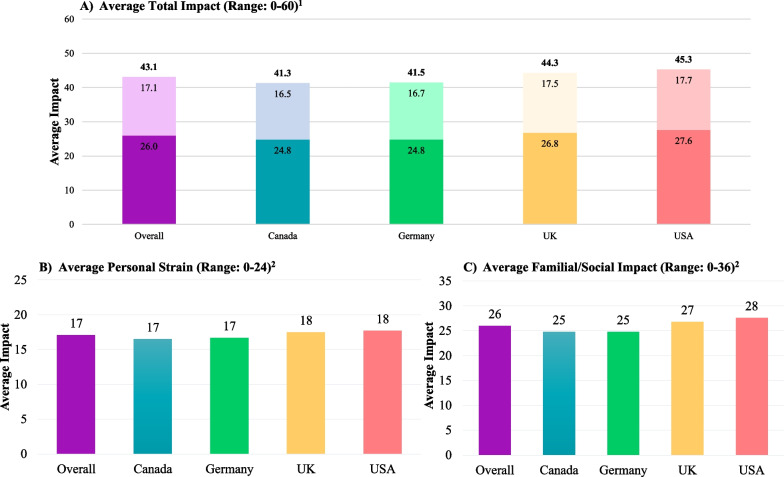
Revised IOFS by Country. BBS: Bardet-Biedl Syndrome; IOFS: Impact on Family Scale. 1. Higher scores indicate that BBS has a greater impact on the family. The bottom bars (dark colors) correspond to the average Familial/Social Impact scores (Range: 0–36) and the top bars (light colors) correspond to the average Personal Strain scores (Range: 0–24). The bolded number at the top of each stacked bar corresponds to the average Total Impact scores (Range: 0–60). 2. Higher scores indicate that BBS has a greater impact on the family

The financial burden due to medical care of patients with BBS was substantial. More than half (53%) of the caregivers reported spending over 5000 out-of-pocket in local currency for medical expenses over the past 12 months; 36% reported spending 1001–5000 out-of-pocket (Fig. 3). Over half (53%) the caregivers reported the financial burden associated with caring for a patient with BBS as “catastrophic” (3%), “significant” (22%), or “moderate” (29%; Fig. 4). Study participants considered BBS to have a moderate-to-high family and social impact as evidenced by an average total RIOFS score of 43 (possible score range: 15–60), with an average personal strain score of 17 (possible score range: 6–24) and an average familial/social impact score of 26 (possible score range: 9–36). Average total impact, personal strain, and familial/social impact scores were similar across countries (Fig. 5).

## Discussion

This cross-sectional survey helps to fill an important gap in the literature by being the first to quantify the burden of caregivers for patients with BBS across four countries and document that this burden is multifaceted and can increase financial strain and barriers to professional work among caregivers, particularly caregivers of young children with BBS. Caregiver responses were mostly similar across countries, although there was some heterogeneity given the differences of the healthcare systems and the extent of availability and accessibility of social supports across countries. Of note, caregiver country of origin was not collected in the survey, which could have an impact on the perception and hence reporting of the burden. Nonetheless, given the rarity of BBS, previous evidence on caregiver burden has been limited to small sample sizes; to that effect the current study makes a novel contribution to the field via its comparably large sample size [10, 11].

These study results substantiate prior qualitative work that found obesity and hyperphagia have a negative impact on the lives of patients with BBS, their caregivers, and their families [15]. One prior interview study of parents of children with BBS found that these parents often experienced distress due to poor awareness of BBS among people in their lives, including healthcare professionals, and the difficulty of coordinating with multiple services to support caretaking activities [10]. Other studies have shown that caregivers of young children with early-onset obesity and BBS often experienced negative social judgment or blame due to their association with a person who was overweight or had obesity, which is an example of weight bias and courtesy stigma [16–18]. A 2015 qualitative study of 28 parents of children with BBS illustrated the impact of such negative social perceptions, as participants reported feeling blamed, devalued and judged by others for their child’s obesity, which resulted in recurrent emotions of anger, frustration, and helplessness among parents [11]. The CARE-BBS study adds to the insight from these studies through a quantification of caregiver burden and its associated negative impacts.

In particular, the IoH and PROMIS questionnaires included in the current study were used to further delineate the negative impacts that caregiving can have on the mental and physical health of caregivers. Results indicate that caregivers experience negative impacts in multiple areas of their lives, including sleep, mood, and emotions. In the current study, caregivers reported a high personal strain and strain on their family, underscoring the substantial and widespread socio-emotional cost of managing obesity and hyperphagia that is characteristically associated with BBS. The reduction in physical and mental wellbeing seen in this study population appears comparable to the burden of caregivers for patients with Prader-Willi syndrome, the most frequently diagnosed cause of syndromic obesity, which is also characterized by severe hyperphagia and associated obesity [19, 20]. These findings suggest that high stress and negative mental and physical consequences of caregiving for patients with severe hyperphagia and obesity such as Prader-Willi syndrome and BBS are common and could result in caregiver burnout, compromising their ability to care for the patient and themselves and leading to further emotional exhaustion and family disruption [21, 22].

The impact of obesity and hyperphagia among patients with BBS on caregiver work productivity and the financial burden related to the costs of medical care and affiliated out-of-pocket expenses was found to be substantial in this study. At an average age of 42 years, caregivers who participated in this study are in their prime age for income-earning (the peak ages are 35 to 44 years as reported by the US Bureau of Labor Statistics) [23]. Caregivers who suffer productivity losses during this key earnings time therefore may further face meaningful income setbacks compared to peers who do not endure similar productivity losses. Caregivers in the current study reported the need to reduce their work hours or quit their jobs altogether, as well as having less productivity and more absenteeism, which reflects the efforts required in providing care to patients with BBS over time and the negative impact it can have on the caregivers’ quality of life. Similar rates of work loss, either temporarily or permanently, were reported among caregivers of those with fragile X syndrome, the world’s most common hereditary cause of intellectual disability [24]. Furthermore, in the 2020 study by Zelihic et al. [10], parents of children with BBS whose workplaces promoted support and coping strategies and enabled fellowship for caregivers reported reduced feelings of isolation within this community. Together with prior research, these findings emphasize the importance of raising awareness of BBS among the general public and healthcare professionals along with strengthening communities and support systems available for caregivers of patients with BBS. Further research is needed to better understand how the availability of different healthcare systems and social support across countries might impact caregiver burden in BBS.

It is worth noting that as a ciliopathy, BBS is a complex condition. Our study focused on quantifying caregiver experiences associated with managing patients’ obesity and hyperphagia; however, the extent of efforts that caregivers putting to support their patients are much greater, for example, due to problems with vision loss, diabetes, renal anomalies, etc [3–5]. There is currently a dearth of literature on burden of caregiving for patients with BBS, other ciliopathies, or syndromes with similar manifestations in hyperphagia and obesity. Our study intended to illuminate the unmet needs related to managing obesity and hyperphagia of patients with BBS. However, this may only represent a portion of the caregiving tasked to these families. For example, one study that characterized burden of caring for children with Joubert syndrome and related disorders (JS/JSRD) found that feeling overwhelmed, physical strain, and family adjustment were the most challenging aspects of caregiving among parents of children with JS/JSRD [25, 26].

Though not a ciliopathy, Prader Willi syndrome is another rare multi-system genetic disorder characterized by obesity and hyperphagia. A study on caregivers of those with Prader-Willi syndrome found that the intensity of hyperphagia was associated with the level of burden that caregivers experiences and patients’ anxiety and behavioral issues further intensified caregiver burden [18] Two studies on caregivers of patients with Prader-Willi syndrome identified challenges arising from intellectual disabilities and higher unplanned healthcare resource use and costs including managing symptoms such as respiratory distress [18, 26].

On the other hand, our study found that the mental health scores of the caregivers were comparable to the general population on the PROMIS Scale. Additionally, though with an overall worse physical health than the general population, about half rated their physical health as good or very good. In fact, only 5% of the caregivers reported a diagnosis of anxiety disorder and less than 5% had depression. Inconsistent findings on mental health of the caregivers of children with rare genetic conditions have been observed in the literature. There was a similar finding in the study of caregivers of children with JS/JSRD: while highly distressed, the majority of the caregivers were not clinically depressed and caregiver burden was not related to disease severity of the children, but rather to parents’ coping skills and level of family functioning [25]. On the contrary, studies assessing the burden among caregivers of patients with Duchenne muscular dystrophy found that the levels of self-reported anxiety and depression were high and were significantly associated with health status of the patients perceived by the caregivers [27, 28]. We speculate that the extent of the burden perceived by the caregivers could be highly subjective given the nature of these rare genetic conditions, the resources available to the families and family dynamics. A high proportion of our study participants had relatively high levels of education, income, and were married or in a domestic partnership. These factors may have contributed to a generally better health status of the participants in our study, and hence our study could have underestimated the true burden of caregiving. Additionally, despite the obvious challenges in caring for a patient with a rare disease, caregivers may have implemented coping mechanisms and taken action to improve their own lives and the lives of the patients they care for, including joining advocacy groups or engaging research activities that may help make a difference for the lives of these children [29]. Further research is much needed to have a full appreciation of the burden and impacts on the lives of the caregivers of patients with rare genetic conditions such as BBS.

### Limitations

A few limitations are to be noted while interpreting the findings from our study. First, all information collected was caregiver-reported, and the reported diagnoses of BBS, obesity, and hyperphagia were not clinically validated. Second, this one-time cross-sectional caregiver survey represents a snapshot in time and cannot be used to draw conclusions about how the burden of caregiving may change over time. A longitudinal study can be valuable to further assess the cumulative burden of the disease or quantify the improvement over time should novel treatments for managing hyperphagia become available. Third, the caregivers taking part in the study were a part of a patient/caregiver panel and may have been more enthusiastic or have more available resources than those who are not involved in such panels. For example, most caregiver participants in our study were college educated (~ 70% with a Bachelors’ or advanced degree) and a higher income than the household average (> 80% with a median household income of ≥ 75,000 in the local currency). It is likely that caregivers with higher incomes/education have more resources available to them to access the needed care support of their patients. Those caregivers who do not have a comparable level of socioeconomic status could encounter additional challenges that are not well reflected in our study. Further research is needed for an in-depth understanding of the full burden of caregiving. Fourth, this study could be subject to selection bias in that only caregivers of patients with BBS who also had obesity and hyperphagia were recruited, although this does represent the large majority of patients with BBS. Finally, the study focused on caregiver burden associated with managing their patients with hyperphagia and obesity. As noted earlier, BBS is a complex medical condition and hence our findings do not reflect the additional burden encountered by the caregivers, for example, caregivers’ effort in caring for other medical aspects of the patients given the BBS’s multifaceted manifestations, and their ability to provide care for the rest of their family.

## Conclusions

This survey conducted across the US, UK, Canada, and Germany is the first to quantify the multifaceted burden of caregivers of patients with BBS, including the daily management of hyperphagia and weight control, caregivers’ ability to work, direct impact on families, and financial strain. These findings provide important information that improves our understanding of how caregivers are impacted while caring for patients with BBS who are suffering from obesity and hyperphagia and highlight the substantial needs for effective interventions to alleviate symptoms and improve the quality of life of patients and their caregivers.

## Supplementary Information

**Additional file 1**. Number of Weight Management Approaches by Country.

**Additional file 2**. Caregiver Employment Changes by Country.

**Additional file 3**. Caregiver Current Employment by Country.

## Data Availability

The data sets used and/or analyzed during the current study are available from the corresponding author on reasonable request.

## Abbreviations

BBS: Bardet-Biedl syndrome
BMI: Body mass index
CARE-BBS: Caregiver burden in Bardet-Biedl syndrome
CRIBBS: Clinical registry investigating Bardet-Biedl syndrome
IoH: Impacts of hyperphagia©
IRB: Independent review board
IWQOL: Impact of weight on quality of life
JS/JSRD: Joubert syndrome and related disorders
NRS: Numeric rating scale
PROMIS: Patient-reported outcomes measurement information system®
RIOFS: Revised impact on family scale©
SD: Standard deviation
UK: United Kingdom
US: United States
WPAI: Work productivity and activity impairment
