# Supplementary material for: Caregiver burden in Bardet-Biedl syndrome: findings from the CARE-BBS study
Source: Orphanet J Rare Dis. 2023 Jul 7;18:181. doi: 10.1186/s13023-023-02692-8 (PMC10327143; doi:10.1186/s13023-023-02692-8)
Supplement: Supplementary file 1 — Additional file 1. Number of Weight Management Approaches by Country. [file 13023_2023_2692_MOESM1_ESM.docx]

***Additional file 1*: *Number of Weight Management*** ***Approaches by Country***
