# Supplementary material for: Caregiver burden in Bardet-Biedl syndrome: findings from the CARE-BBS study
Source: Orphanet J Rare Dis. 2023 Jul 7;18:181. doi: 10.1186/s13023-023-02692-8 (PMC10327143; doi:10.1186/s13023-023-02692-8)
Supplement: Supplementary file 2 — Additional file 2. Caregiver Employment Changes by Country. [file 13023_2023_2692_MOESM2_ESM.docx]

***Additional file 2. Caregiver Employment Changes by Country***

|  | **Overall N = 200** | **Canada N = 48** | **Germany N = 50** | **UK N = 49** | **US N = 57** |
| --- | --- | --- | --- | --- | --- |
| **Proportion of participants who experienced an employment event** | | | | | |
| Had to switch jobs, n (%) | 17 (8.5) | 1 (2.1) | 3 (6.0) | 9 (18.4) | 4 (7.5) |
| Time from diagnosis of BBS to employment (months), mean ± SD | 4.8 ± 18.3 | 13.0 ± - | 2.7 ± 43.7 | 4.4 ± 9.2 | 5.3 ± 14.4 |
| Had to temporarily stop working or went on leave, n (%) | 37 (18.5) | 8 (16.7) | 6 (12.0) | 14 (28.6) | 9 (17.0) |
| Time from diagnosis of BBS to employment (months)^1^, mean ± SD | 6.1 ± 25.9 | 5.5 ± 4.8 | 25.7 ± 25.1 | 4.8 ± 4.9 | -5.5 ± 48.6 |
| Had to permanently stop working/retire early, n (%) | 29 (14.5) | 8 (16.7) | 10 (20.0) | 3 (6.1) | 8 (15.1) |
| Time from diagnosis of BBS to employment (months)^1^, mean ± SD | 7.6 ± 32.5 | 4.6 ± 1.8 | 17.9 ± 43.4 | 16.7 ± 14.0 | -3.1 ± 41.1 |
| Had to reduce work hours, but could keep working, n (%) | 39 (19.5) | 4 (8.3) | 11 (22.0) | 14 (28.6) | 10 (18.9) |
| Time from diagnosis of BBS to employment (months)^1^, mean ± SD | 7.0 ± 23.2 | 17.7 ± 43.6 | 1.3 ± 16.4 | 13.8 ± 25.9 | -1.4 ± 7.5 |
| Had to change the activities performed at work, but could keep working, n (%) | 13 (6.5) | 3 (6.3) | 2 (4.0) | 2 (4.1) | 6 (11.3) |
| Have not been as productive, n (%) | 9 (4.5) | 0 (0.0) | 3 (6.0) | 4 (8.2) | 2 (3.8) |

**Abbreviations**: BBS: Bardet-Biedl Syndrome; SD: standard deviation.

**Note**: 1. A negative mean reflects that caregivers often experienced the employment event before a confirmed diagnosis.
