# Supplementary material for: Caregiver burden in Bardet-Biedl syndrome: findings from the CARE-BBS study
Source: Orphanet J Rare Dis. 2023 Jul 7;18:181. doi: 10.1186/s13023-023-02692-8 (PMC10327143; doi:10.1186/s13023-023-02692-8)
Supplement: Supplementary file 3 — Additional file 3. Caregiver Current Employment by Country. [file 13023_2023_2692_MOESM3_ESM.docx]

***Additional file 3. Caregiver Current Employment by Country***

|  | **Overall N = 242** | **Canada N = 62** | **Germany N = 61** | **UK N = 59** | **US N = 60** |
| --- | --- | --- | --- | --- | --- |
| **Proportion of participants reporting any active employment (full-time, part-time or self-employed)** | | | | | |
| Any employment | 183 (75.6) | 44 (71.0) | 48 (78.7) | 47 (79.7) | 44 (73.3) |
| **Employment type** | | | | | |
| Self-employed | 11 (4.5) | 2 (3.2) | 3 (4.9) | 4 (6.8) | 2 (3.3) |
| Paid full-time (40 hours/week) employee | 142 (58.7) | 37 (59.7) | 41 (67.2) | 31 (52.5) | 33 (55.0) |
| Paid part-time (temporary or seasonal) employee | 33 (13.6) | 5 (8.1) | 6 (9.8) | 13 (22.0) | 9 (15.0) |
| Unemployed and looking | 8 (3.3) | 2 (3.2) | 2 (3.3) | 1 (1.7) | 3 (5.0) |
| Unemployed and not looking | 18 (7.4) | 5 (8.1) | 2 (3.3) | 3 (5.1) | 8 (13.3) |
| Retired | 3 (1.2) | - | 3 (4.9) | - | - |
| Homemaker/housewife/  househusband | 30 (12.4) | 11 (17.7) | 6 (9.8) | 8 (13.6) | 5 (8.3) |
